# 

**Published:** 1889-08-10

**Authors:** 


### The Students' Column.

### EDINBURGH MEDICAL EXAMINATIONS.

The following is the official list of candidates who passed the Second Professional Examination in Medicine at Edinburgh University:

John Anderson, R. J. Ashton (with distinction), Hugh Bennett, R. J. A. Berry, J. W. Bone (with distinction), H. J. M. Buist, W. M. Cairns, K. M. Cameron, J. J. W. Campbell, Charles Cochrane, R. P. Cockburn, D. B. Crerar, G. D. Darlington, Arthur Dennison, H. R. L. Davis, C. J. W. Dixon (with distinction), D. P. M. Farquharson, F. T. Foster, E. J. Fox, R. A. Fryer, E. B. Garland, P. F. Grant, Henry Goudie, Frederick Gourlay, G. C. Greaves, F. T. Griffin, T. A. Green, R. E. Harrold, H. H. Hearsey, G. E. Helme, H. M. Hewer, A. G. Horden, J. V. Hulme, John Joakim, J. M. M. Kay, C. B. Ker, Robert Lamb, M.A., E. P. T. von Landsberg, H. E. Lee, R. E. Little, John Livingstone, R. M. Lyon, James MacBride, J. G. Macdonald, James Macfarlane, H. M. MacGill, Wakefield MacGill, T. G. M'Kellar, H. J. Mackenzie, H. V. M'Kenzie, John Maclaren, J. D. Maclean, W. B. Macleod, Charles Macmaster, C. B. Martin, H. F. Malabre, George Melville, J. Adie Menzies, A. S. Miller, Alexander Mitchell, T. W. Mitchell, E. C. Moore, C. A. Morgan, Robert Muir, J. A. Murison, John Newington, P. A. Nightingale, N. G. Noble, C. M. Ormsby, John Panton, Wm. Paul, E. R. Parry, R. L. Price, Harry Rainy (with distinction), J. L. Reed, E. H. Ridley, J. H. Roberts, J. C. Robertson, David Rorie, Robert Rosie, Wm. Sanders, Walter Sansom, P. W. Schmidt, John Sharp, William Simmers, F. M. T. Skae, Thomas Stodart, Archibald Stodart-Walker, Robert Strachan, Charles Stuart, David Stuart (with distinction), William Thyne, J. C. Tosswell, A. K. Thomson, L. J. Weatherbe, J. C. Whyte, George Wilson, O. R. M. Wood, James Younan, R. J. E. Young.

### SPECIAL ANNOUNCEMENT.

### MISS FLORENCE NIGHTINGALE.

The next (September) monthly part of The Hospital will be accompanied by a portrait of Miss Nightingale, which has been reproduced at great cost and perfection; and it will at last be possible for every member of the nursing profession to possess the likeness of one who, by a life of self-sacrifice and philanthropic labour, has raised herself to pre-eminence among women. The monthly number will be issued at sixpence as usual, but orders should be sent early.

We regret that, owing to an accident which has happened to the plate at the collotyping works where this portrait is being produced, we were compelled to postpone its publication to the next (September) monthly part. Though the delay thus caused is much to be regretted, there is consolation in the knowledge that a really excellent portrait of Miss Nightingale will be within reach of our readers a few weeks hence. The biographical sketch appeared in our issue of July 27.

### NOTICE TO CORRESPONDENTS.

All MS., Letters, Books for Review, and other matters intended for the Editor, should be addressed The Editor, The Lodge, Porchester Square, London, W.

**Notice to Advertisers and Subscribers.**

All Advertisements, Orders for Copies of The Hospital, and Business Communications should be addressed to The Publisher, *not to the Editor*, The Hospital, 139-140, Salisbury-court, Fleet-street, E.C.

Vol. V., now ready, 4s., post free. Cases for binding, may be had of the Publisher, at 1s. 6d. each.

**To Residents, Secretaries, and Matrons.**

The Editor will be glad to have the Regulations and each Report as issued by Asylums, Hospitals, Convalescent and Nursing Institutions, as well as those of other Charities, and an early copy in duplicate of all Appeals and Festival Notices, etc., addressed to *The Editor, The Lodge, Porchester-square, London, W.* Any superintendent, secretary, matron, or other chief official who regularly sends such information may be registered, on application to the Publisher, to receive free of cost a cover for binding The Hospital in half-yearly volumes.

### Amusements and Relaxation.

### Second Quarterly Word Competition Commenced July 6th, ends September 28th.

Three Prizes of 15s., 10s., 5s., will be given for the largest number of words derived from the words set for dissection.

Proper names, abbreviations, foreign words, words of less than four letters, and repetitions are barred; plurals, and past and present participles of verbs, are allowed. Nuttall's *Standard* dictionary only to be used.

N.B.—Word dissections must be sent in WEEKLY, arranged alphabetically, with correct total affixed.

The word for dissection for this, the *Sixth* week of the quarter, being

"SPITHEAD."

**Results of Fourth Week.**

| Names. | Aug. 2nd. | Totals. | Names. | Aug. 2nd. | Totals. |
|---|---|---|---|---|---|
| Nurse Duty | 22 | 259 | Rover | — | 165 |
| N'importe Qui | 20 | 229 | Leirion | 19 | 195 |
| Jumps | — | 30 | Matron | 17 | 149 |
| Tinie | — | 186 | F. C. J. | 18 | 181 |
| Patience | 22 | 249 | Percy Verance | — | 18 |
| Amicus | 21 | 246 | Gleniffer | — | 175 |
| Broxbourne | 22 | 252 | Gypsye | 19 | 216 |
| Speedwell | 20 | 236 | Ladyr | — | 127 |
| Sister | 22 | 243 | A. B. | — | 170 |
| Lightowlers | 20 | 250 | Essie | 19 | 197 |
| Lauriston | 19 | 246 | W. G. R. | 16 | 196 |
| Rattler | — | 28 | Cowboy Bill | — | 179 |
| Paignton | — | 179 | M. L. A. | — | 117 |
| Fassifern | — | 141 | Little Tuppy | — | 110 |
| W. R. E. | 21 | 248 | Embryo | — | 56 |
| Esperance | 21 | 250 | K. Jarman | — | 43 |
| M. W. | 21 | 255 | Reveal | — | 196 |
| Isabel | 21 | 236 | Jenny Wren | 20 | 183 |
| Buxton | 17 | 167 | Condorcet | 15 | 209 |
| Nux Vom | 22 | 235 | Electrician | 18 | 45 |
| Qu'appelle | 18 | 224 | Pearl | — | 38 |
| Spes | — | 24 | R. W. | — | 43 |
| Secnarf | 17 | 229 | Nurse Amie | — | 89 |

**Notice to Correspondents.**

N.B.—All letters referring to this page which do not arrive at 139 and 140, Salisbury-court, London, E.C., by the *first post on Thursdays*, and are not addressed PRIZE EDITOR, will in future be disqualified and disregarded.

Competitors can enter for *all* quarterly competitions, but no competitor may take more than *two quarterly prizes* during the year.

### Scraps and Gleanings.

There is a water famine in Vienna.

Deaths from drowning have risen to 10 a week.

Dr. Burney Yeo writes in the *Nineteenth Century* on "Change of Air."

Antipyrin and lanoline have been added to the Austrian pharmacopœia.

A Pasteur Institute is to be founded at Rome by the Municipa Council.

Professor Gamgee has severed his connection with St. George's Hospital.

The *Cornhill Magazine* contains a gruesome article on "Curiosities o Leperdom."

Radcliffe Infirmary has received £50 from the Oxford Hospital Sunday Fund.

The receipts from Hospital Saturday at Bolton reach the handsome total of £1,482.

The Homerton Fever Hospital has a plentiful supply of diphtheria cases just now.

Much sickness prevails amongst our troops in Upper Burmah; chiefly fever and scurvy.

Pasteur is again unsuccessful; a young lad of 13, treated by him, has died of hydrophobia.

Hospital Saturday in Coventry resulted in £62; larger by £4 than last year's collection.

The West London Hospital lately benefited by a flower service he at St. Paul's, Brentford.

Mr. E. B. Whitcombe, of Birmingham Asylum, has been electe Ingleby lecturer for 1890.

Madame Edouard Andre has sold all her jewels, and with the price started a dispensary in Paris.

Sir Morell Mackenzie and Sir W. W. Hunter both contribute to the current *Contemporary Review.*

Sunday Promenade Concerts are held at Northampton, and the proceeds given to the hospital.

Plymouth death-rate has been high lately, 28·0 per cent.; whilst Huddersfield only registers 10·7.

Dr. Macan, of the Rotunda Hospital, has been elected professor of midwifery in Dublin School of Physic.

Mr. M. Campbell, once of Greenock, late of Montreal, has left 1,500 dollars to the Montreal General Hospital.

Thomas Clarke, M.D., has published a small book called "The Fate of the Dead: An Address to Laymen."

The Hon. Dr. Ross, ex-premier, has been elected president of the Quebec College of Physicians and Surgeons.

The Anchor Brewery Band plays sometimes in the grounds of the Norwich Hospital. Other bands please note.

The proposed Convalescent Home for North of England Friendly Societies will probably be at Morecambe or Grangover Sands.

Coventry Provident Dispensary is blamed for the rapid spread of infectious diseases in that town. Sick people meet and huddle together there.

The Society of Apothecaries gives notice that in future every candidate for the L.S.A. must produce evidence of having operated on dead bodies.

The Queen wore at the Royal wedding the necklace and earrings given to her Majesty by the women of England as part of their Jubilee offering.

It has been reported to the Committee of Works of the St. George's, Hanover-square, Vestry, that an old army pensioner has died in St. George's Hospital from leprosy.

Mdlle. Blanche Edwards, M.D., spoke at the Paris Congress on the desirability of a physical education for girls. The French schools are particularly defective in this respect.

The Committee of the New Hospital for Women wish to purchase their new site, which will need from £7,000 to £8,000. Towards this end a bazaar will be held in the spring.

Madame Dejerine read her thesis on "Nervous Disorders in General, and Saturnine Varieties of Paralysis and Atrophy in Particular," before the leading physicians of Paris on July 27th.

Brown-Seguard's Elixir of Life is beaten by a spring in Nevada, which it is said to have turned a septuagenarian nigger into a gay and lively youth. They do these things best in America.

The Cameron prize in therapeutics has been awarded to M. Pasteur, of Paris, in recognition of the high importance and great value to practical therapeutics of the treatment of hydrophobia discovered by him.

Prince Bismarck's special medical attendant, Professor Schweninger, who cured the Chancellor of his too pronounced tendency to stoutness, is, by particular desire of the Sultan, about to instruct two Turkish physicians in his special method of treatment.

Three men, hailing individually from Wellington College, Berkshire, from Portland, Weymouth, and from Shepherd's Bush, each of whom has been bitten by a mad dog, were sent to M. Pasteur's institution in Paris, at the expense of the Lord Mayor's fund.

During the excessively hot weather numerous captures of stray dogs have been made in the streets of Birmingham. The animals have been cremated at one of the Corporation furnaces at the rate of fifty a day. The dogs are previously poisoned with prussic acid.

An ironworker, named Bleicher, living at Iserlohn, has had extracted from his left leg a bullet which he received from a French chassepot rifle in the battle of Mars-la-Tour, in 1870. Since that time he has followed his calling with the bullet in the limb. He is now progressing favourably.

A Zanzibar telegram says that fever is prevalent there and among the fleets, the English apparently suffering most. On the "Agamemnon" alone eighty are sick, including seven officers, out of a total complement of 400. The deck has been transformed into a hospital, and hammocks have been slung everywhere.

